# Boosting the Motor Outcome of the Untrained Hand by Action Observation: Mirror Visual Feedback, Video Therapy, or Both Combined—What Is More Effective?

**DOI:** 10.1155/2018/8369262

**Published:** 2018-04-10

**Authors:** Florian Bähr, Alexander Ritter, Gundula Seidel, Christian Puta, Holger H. W. Gabriel, Farsin Hamzei

**Affiliations:** ^1^Department of Sports Medicine and Health Promotion, Friedrich Schiller University Jena, Jena, Germany; ^2^Section of Neurological Rehabilitation, Hans Berger Department of Neurology, Jena University Hospital, Jena, Germany; ^3^Department of Neurology, Moritz Klinik Bad Klosterlausnitz, Bad Klosterlausnitz, Germany

## Abstract

Action observation (AO) allows access to a network that processes visuomotor and sensorimotor inputs and is believed to be involved in observational learning of motor skills. We conducted three consecutive experiments to examine the boosting effect of AO on the motor outcome of the untrained hand by either mirror visual feedback (MVF), video therapy (VT), or a combination of both. In the *first* experiment, healthy participants trained either with MVF or without mirror feedback while in the *second* experiment, participants either trained with VT or observed animal videos. In the *third* experiment, participants first observed video clips that were followed by either training with MVF or training without mirror feedback. The outcomes for the untrained hand were quantified by scores from five motor tasks. The results demonstrated that MVF and VT significantly increase the motor performance of the untrained hand by the use of AO. We found that MVF was the most effective approach to increase the performance of the target effector. On the contrary, the combination of MVF and VT turns out to be less effective looking from clinical perspective. The gathered results suggest that action-related motor competence with the untrained hand is acquired by both mirror-based and video-based AO.

## 1. Introduction

Research of the past years clearly demonstrated that action observation (AO) is an effective method to boost motor skill learning (see [1–4] for reviews on observational learning). In fact, the leading advantage of the AO concept is the boosting effect on motor performance before the actual execution takes place. This is because a video-depicted action conveys a visuomotor and sensorimotor information to the observer that contains on the one hand the goal of the action and on the other hand how this action is being performed accurately [5–7]. AO is therefore a promising method in the field of neurorehabilitation to improve the motor and the functional outcome of stroke patients (see [8] for a review).

However, basic concepts of the applied AO interventions in neurorehabilitation stem from findings of neurophysiological studies on *mirror neurons* which explained the promoting effect of AO on action execution for the first time [9–11]. Furthermore, execution and observation of a goal-directed motor act excite the same mirror neuron population [12]. The main hypothesis about the *mirror neuron mechanism* postulates that the goal of an action links the performing actor with the observer by stimulating a reenactment of similar embodied action representations that are already stored in the motor repertoire of the observer [13, 14]. Further studies demonstrate that the mirror neuron mechanism is multimodal and not only triggered by a visual stimulus. Instead, it can be triggered when merely an action is presented acoustically [15], when some events of an action remain hidden [16], and when different actions were required to reach the same goal [17]. Mirror neurons were found in several brain areas of nonhuman primates: the premotor cortex (PMC), the inferior parietal lobule (IPL), and the superior temporal sulcus (STS). These aforementioned subareas are component units of what is functionally summarized as the *mirror neuron system* (MNS) [18]. Recent functional magnetic resonance imaging (fMRI) studies suggested that a comparable network also exists in humans which is being termed *action observation network* (AON) [19–22]. In this regard, a large-scale brain fMRI study and two meta-analyses reported a robust overlapping network that comprises areas of IPL and the inferior frontal gyrus (IFG) including the Broca's area that was also activated during AO and an immediate execution of observed actions [21–23]. Further studies supposed that the AON is primarily involved in observational learning of new motor skills [24, 25] and facilitates skill acquisition after stroke [26]. Accordingly, Fadiga et al. demonstrated that AO facilitates the primary motor cortex (M1), which in turn excites the same muscles in the same dynamics responsible for the execution of the observed action [27]. For example, Calvo-Merino et al. found increased fMRI activation in the AON when highly experienced dancers observed their characteristic embodied dance movements compared to unfamiliar movements of another dance style [28]. Additionally, Stefan et al. showed that AO facilitates learning of unfamiliar thumb movements by driving the formation of new motor memories inside M1, which are comparable to those acquired by physical practice [29]. From a clinical point of view, it is particularly interesting that a comparable effect of AO on motor learning was also demonstrable on older adults [30], and on stroke patients [31]. In a pilot study, employing the newly introduced *video therapy* (VT), Ertelt et al. showed that AO improves motor recovery after stroke. The combination of video observation and immediate execution of the observed movements with the paretic hand resulted in a highly significant improvement of motor performance compared to the execution after observing geometric symbols [26]. AO thus opens the opportunity to boost functional recovery without necessarily moving the paretic hand. Interestingly, exactly this basic concept has already been successfully applied in stroke rehabilitation using the so-called *mirror visual feedback* (MVF) [32]. Here, patients sit in front of a mirror placed along the midsagittal plane between both arms. While looking continually into the mirror, patients perform a motor task with their nonaffected arm. This creates the optical illusion that the paretic arm is performing the task. Remarkably, clinical studies using MVF reported improved motor performance on the untrained paretic hand [33–35]. Therefore, further studies assume that the MVF effect on the untrained hand is directly related to the activation of the AON as a result of inputs received via the mirror while observing one's own actions [32, 36–42].

MVF and VT provide inputs of an action (mirrored or displayed) that are matching the actual execution with the untrained hand. It is likely that these inputs are processed via the AON to build up a task-related motor schema for the target effector. Therefore, our primary aim was to examine the boosting effect of action observation (AO) on the motor outcome of the untrained hand by means of mirror visual feedback (MVF), video therapy (VT), or a combination of both. Our main motivation for this study was the evaluation of the effectiveness of the deployed methods with the prospect of application in stroke therapy on patient with a severe paresis. Thus, two questions arise which may be crucial for an application of MVT and VT. The first question examines what boosting effect AO has on the motor result of the untrained hand in both conditions (MVF and VT). To clarify this question, we conducted two experiments. The *first* experiment scrutinized the effect of AO after training with MVF compared to training without mirror feedback as already demonstrated in a previous study [37]. We suggested that motor outcome of MVF is superior due to the AO during the training. By using the same tasks, in the *second* experiment, we examined the effect of AO after observing action-related video clips (VT) compared to non-action-related animal video clips. In this regard, we expected that the outcome for VT is superior because of the AO during the training. The second question then investigates the possibility of an increased training effectiveness by combining MVF and VT. Therefore, in the *third* experiment, we examined the effect of additional AO on motor outcome of the untrained hand. Participants first observed action-related video clips (VT) followed by either training with MVF or training without mirror feedback. In consequence, we presumed that the combination of training with VT and MVF is more effective due to the additional AO.

## 2. Materials and Methods

### 2.1. Participants

A total of 60 right-handed (according to the Edinburgh handedness inventory [43]) healthy volunteers (29 females; age 21–27 years, *M* = 23.38 ± 1.58 years) participated in this study. No one had a history of brain trauma or other disease that alters the brain. Exclusion criteria were drug use and musculoskeletal or neurological diseases. Volunteers gave informed consent before participating in this study, which was approved by the Ethics Committee of the Friedrich Schiller University Jena and conformed to the standards set by the Declaration of Helsinki (1964).

### 2.2. Experimental Protocol

At baseline (*pre*), all participants initially performed a standardized motor skill test including five tasks [37, 38, 44]. Each participant performed the respective tasks with the left (test) hand for exactly two minutes, which amounts to a total testing time of 10 minutes. The five tasks were performed as follows: (i) *Marbles*: participants used a teaspoon to move marbles from one bowl to another. Marbles successfully moved into the new bowl were counted. (ii) *Nine-hole peg test (NHPT)*: participants were asked to remove a peg out of the board and place it onto a predefined position on a desk before they return the previously removed peg into the board. Returned pegs were then counted. (iii) *Cards*: participants were asked to draw cards from a stack and turn them before they stacked them one above another onto a predefined position on a desk. The cards of the new stack were then counted. (iv) *Pick-a-stick*: a number of sticks were positioned one after another in front of the participant parallel to the edge of a desk on a predefined position. At first, participants were asked to take the nearest stick in front of them. With the help of the tip of this stick, they were required to lightly move the following stick into their direction before they are to take it, too. Without putting away the supporting stick, participants were asked to place the other stick into a drinking glass located atop the positioned sticks on the desk. Again, sticks successfully placed into the drinking glass were then counted. (v) *Rubber band*: participants were asked to take a rubber band from a stack and to unroll it along the outside of a drinking glass. The successfully unrolled rubber bands were then counted. Furthermore, participants were encouraged to execute the given tasks quickly and to remain focused on their task while an instructor sat next to them.

**Figure 1:**
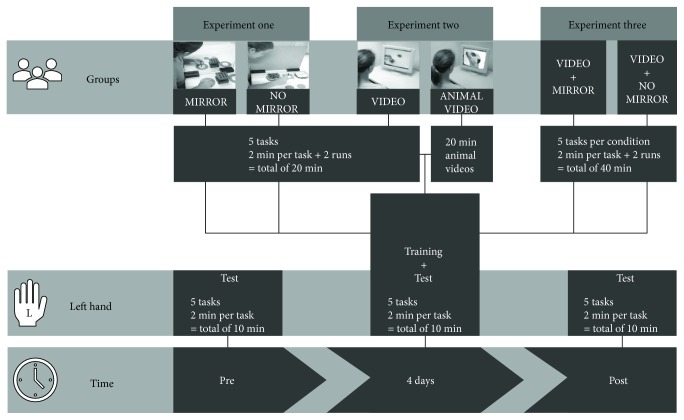
Schedule of the study. On the bottom, from left to right: timeframe from *pre* (baseline) to *post*. Thereover, from left to right: scope of the content at the points in time. Uppermost: group conditions within the three experiments and their training protocol.

Following the initial baseline (*pre*), participants started a training session where they trained each task for two minutes in two runs with a three-minute break between both runs (the total training time for the five tasks was 20 min). At the end of each training session, the left hand was tested again. The purpose of the daily testing was to assess possible learning and ceiling effects. After four days of training (*post*), all participants performed the same standardized test with the left hand (tasks and conditions were the same as on the first day of the study; see Figure 1).

### 2.3. Experiments

#### 2.3.1. First Experiment: Effect of AO by Training with MVF Compared to Training without Mirror Feedback

In the *first* experiment, 20 participants were randomly assigned to two groups. Participants from the mirror training group MIRROR (*n* = 10, six females) trained while they were continually looking into a mirror placed along the midsagittal plane between their arms. In contrast, participants from the NO MIRROR group (*n* = 10, five females) trained while they were looking continually at their training hand. For this purpose, a board with the same dimensions as the mirror was placed along the midsagittal plane between their arms. Participants from both groups could not see their test hand and were instructed to refrain from any movements with this hand.

#### 2.3.2. Second Experiment: Effect of AO by Training with Action-Related Video Clips (VT) Compared to Non-Action-Related Animal Video Clips

In the second experiment, 20 participants were randomly assigned into two groups. Participants from the video training group VIDEO (*n* = 10, four females) observed prerecorded video tapes that contain the respective tasks of daily training sessions performed with the test hand. Video tapes showed the actor from the first-person perspective. The number of videos displayed during training sessions corresponded to the mean amount of action executions per task of the group NO MIRROR from the first experiment. The number of videos was then accordingly adjusted to each training session. Participants from the animal video group ANIMAL VIDEO (*n* = 10, five females) observed non-action-related animal video tapes in their training sessions (in the same amount of time as in the VIDEO group). Video training sessions in both groups had been conducted in the same period of time scheduled for the training sessions in the groups of the *first* experiment.

#### 2.3.3. Third Experiment: Effect of Additional AO on Motor Outcome of the Untrained Hand

In the *third* experiment, 20 participants were randomly assigned into two groups. Participants from the mirror training group with additional video training, that is, VIDEO + MIRROR (*n* = 10, four females) first underwent the VIDEO procedure (as described in the *second* experiment) and then the MIRROR procedure (as described in the first experiment). Participants of the no mirror training group with additional video training, that is, VIDEO + NO MIRROR (*n* = 10, five females) first carried out the VIDEO procedure (as described in the *second* experiment) and secondly the NO MIRROR procedure (as described in the first experiment). Thus, these training sessions lasted twice as long as in the *first* and *second* experiment.

### 2.4. Data Analysis

The result of the untrained hand in each of the five respective tasks (i.e., marbles, nine-hole peg test, cards, pick-a-stick, and rubber band) of each participant was summed up and then divided by the number of tests. This average sum was then defined as the mean score of the overall test result of the untrained hand (*M*). The calculated mean scores of pre- and post-measurements (*M*_pre_, *M*_post_) were compared to compute the score difference (*ΔM*) for each participant (*ΔM = M*_post_ − *M*_pre_). Statistical calculations were carried out using IBM SPSS Statistics 23 (IBM, Armonk, NY, USA) and GraphPad Prism 7 (GraphPad Software, La Jolla, California, USA). Normal distribution was determined by D'Agostino & Pearson omnibus normality test. Levene's test was applied to assess the equality of variances between the groups for each experiment. In order to test the differences between the groups in each experiment, a two-way analysis of covariance (ANCOVA; between-subjects factor *group*, within-subjects factor *ΔM*, and covariate *M*_pre_) was performed as proposed by Atkinson and Batterham [45]. We considered values of *p* < 0.05 to be statistically significant. Additionally, we calculated the effect size Glass' delta (*Δ*) of each experimental condition (MIRROR, NO MIRROR, VIDEO, VIDEO + MIRROR, and VIDEO + NO MIRROR) on the left test hand compared to the control condition (ANIMAL VIDEO) from the *second* experiment as proposed by Hedges and Olkin [46]. Cohen defined that *Δ* ≤ 0.2 indicates a small effect, *Δ* ≤ 0.5 indicates a medium effect, and *Δ* ≤ 0.8 indicates a large effect [47].

## 3. Results

### 3.1. First Experiment

**Figure 2:**
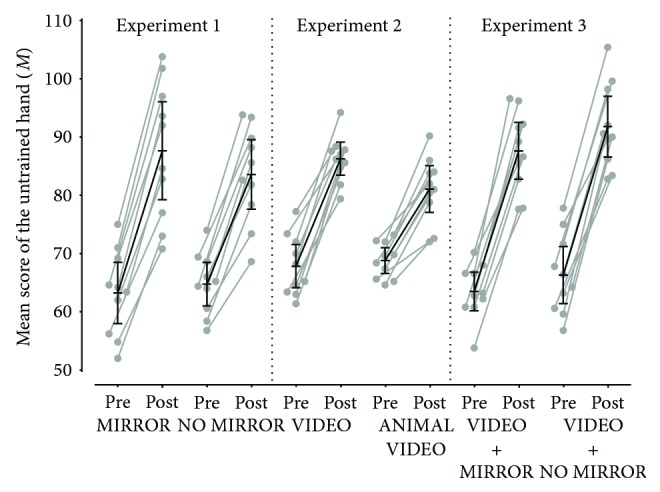
Mean score of the overall test result of the untrained hand (*M*) to each point of measurement (pre, post; individual values, mean, and 95% CI) of each experimental condition (MIRROR, NO MIRROR, VIDEO, TEST, VIDEO + MIRROR, and VIDEO + NO MIRROR) assigned to the corresponding experiment which are separated from each other by the dotted line. Mean score of the overall test result of the untrained hand (*M*) was calculated as follows. The test result of the untrained hand in each of the five respective tasks (i.e., marbles, nine-hole peg test, cards, pick-a-stick, and rubber band) was summed up for each participant and then divided by the number of tests.

**Figure 3:**
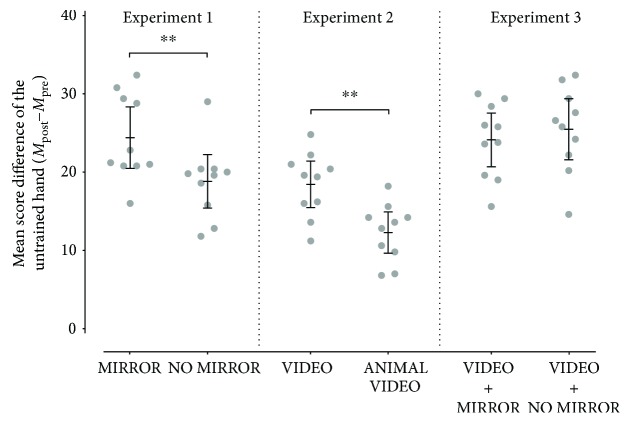
Mean score difference of the overall test result of the untrained hand to each point of measurement (*ΔM* (*M*_post_ − *M*_pre_); individual values, mean, and 95% CI) of each experimental condition (MIRROR, NO MIRROR, VIDEO, TEST, VIDEO + MIRROR, and VIDEO + NO MIRROR) assigned to the corresponding experiment which are separated from each other by the dotted line. Asterisks (^∗∗^) indicate the significant main effect of the factor group of *p* < 0.01.

Analyzing the mean score of the overall test result of the untrained hand (*M*) of the groups MIRROR (*M* = 24.4, 95% CI 20.48–28.31) and NO MIRROR (*M* = 18.82, 95% CI 15.39–22.24) after four days of training, ANCOVA with the factors group, mean score difference (*ΔM*), and the covariate mean score at baseline (*M*_pre_) revealed a significant main effect for the factor group (*F*_1,17_ = 10.08, *p* = 0.006, *η*^2^ = 0.372). This main effect resulted from the overall higher mean score of the group MIRROR compared to the group NO MIRROR (see Figures 2 and 3).

### 3.2. Second Experiment

Analyzing the mean score of the overall test result of the untrained hand (*M*) of the groups VIDEO (*M* = 18.44, SD = 4.14) and ANIMAL VIDEO (*M* = 12.28, SD = 3.69) after four days of training, ANCOVA with the factors group, mean score difference (*ΔM*), and the covariate mean score at baseline (*M*_pre_) revealed a significant main effect for the factor group (*F*_1,17_ = 11.57, *p* = 0.003, *η*^2^ = 0.405). This main effect resulted from the overall higher mean score of the group VIDEO compared to the group ANIMAL VIDEO (see Figures 2 and 3).

### 3.3. Third Experiment

No significant differences were found between the mean score of the overall test result of the untrained hand (*M*) of the groups VIDEO + MIRROR (*M* = 24.12, SD = 4.79) and VIDEO + NO MIRROR (*M* = 25.48, SD = 5.45) after four days of training. ANCOVA with the factors group and mean score difference (*ΔM*) including the covariate mean score at baseline (*M*_pre_) revealed no significant main effect (*F*_1,17_ = 5.61, *p* = 0.464, *η*^2^ = 0.032) (see Figures 2 and 3).

### 3.4. Effect Sizes

**Figure 4:**
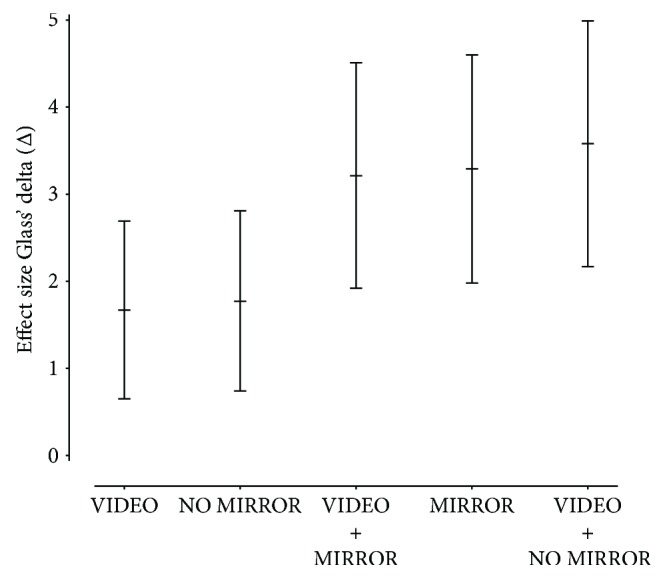
Effect size Glass' delta (*Δ* and 95% CI) of the experimental conditions VIDEO, NO MIRROR, VIDEO + MIRROR, MIRROR, and VIDEO + NO MIRROR of the untrained hand after four days of training.

Calculation of the effect size Glass' delta (*Δ*) of the experimental conditions after four days of training revealed the largest effect of the untrained hand for VIDEO + NO MIRROR (*Δ* = 3.58, 95% CI 2.17–4.99), followed by MIRROR (*Δ* = 3.29, 95% CI 1.94–4.63), VIDEO + MIRROR (*Δ* = 3.21, 95% CI 1.89–4.54), and NO MIRROR (*Δ* = 1.77, 95% CI 0.74–2.81). The smallest effect was found for the condition VIDEO (*Δ* = 1.67, 95% CI 0.65–2.69) (see Figure 4).

## 4. Discussion

The primary aim of the present study was to examine the boosting effect of action observation (AO) on the motor outcome of the untrained hand by means of mirror visual feedback (MVF), video therapy (VT), or a combination of both. In our *first* experiment, we confirmed the boosting effect of AO during MVF by means of previously evaluated tasks [22, 40, 48]. Motor outcome of the untrained hand was greater after training with MVF compared to training without mirror feedback. With our findings from the *second* experiment, we extend the spectrum of application for the tasks to the possibility of using them in a video training protocol (VT). Here, we demonstrated a boosting effect on motor outcome of the untrained hand by mere AO in comparison to watching non-action-related animal video clips. Eventually, in our *third* experiment, we combined MVF and VT for the first time and demonstrated that doubling AO has no additional boosting effect.

### 4.1. The Boosting Effect of Action Observation on the Motor Outcome of the Untrained Hand during Both Mirror Visual Feedback and Video Therapy

The reported positive effect by MVF on the performance of the untrained hand from our *first* experiment is well established and already demonstrated on healthy adults using the same motor tasks [37], and on patients suffering from stroke [33–35]. Several studies thus infer that this effect is based on inputs received by AO during the observation of one's own action in the mirror [32, 36, 37, 39, 40]. This notion is supported by findings that show human premotor and parietal regions of the AON becoming active during both execution and observation of similar motor acts [21, 22, 49]. The same regions also exhibit a strong homology to MNS-associated regions in nonhuman primates [50]. However, activation of these regions within the AON increases as a function of motor competence linked to the observed action [28, 51–54]. By means of an optical illusion, the received mirror-based inputs of AO may feed a motor schema with visuomotor information that is consistent with the execution of the resting target effector during MVF training (MIRROR). In order to benefit from AO during MVF, control signals for the muscles within the motor schema are possibly transformed by the received corresponding visuomotor information [5, 27, 29]. This constitutes a sharp difference to training without a mirror (NO MIRROR), since here, visuomotor information of the actual training hand is being processed. We therefore evaluate this information to be rather “inconsistent” with respect to the passive test hand because there is no transfer to the muscles of the target effector. We assume that the lack of control signals for the muscles of the target effector during training without a mirror may be a possible explanation for the significant difference to training with MVF, since these signals are processed by AO.

Accordingly, results from our *second* experiment clearly indicate the positive effect of mere AO on the motor outcome of the untrained hand when participants watch video clips that show the target effector performing the assigned tasks (VIDEO; see Figure 3). In contrast to non-action-related animal videos (ANIMAL VIDEO), action-related video clips provide visuomotor inputs that contain consistent information about the actual execution of the tested motor tasks with the target effector. Our results correspond with previous findings on AO, demonstrating that even mere short-term observational practice mediates visuomotor information of the observed action that immediately improves the motor outcome of both hands, regardless of which hand was observed [5, 6]. However, it remains unclear how the visual information about an action is linked to the brain of the observer [55]. One idea is that the sensorimotor system is activated during AO [56]. Current studies further support this idea by demonstrating substantial anatomical projections from the primary and secondary somatosensory cortices (S1, S2) to the intraparietal area (AIP) [57–59] as well as projections from parietal areas to M1 and the PMC via S1 [60]. A recent study postulates that the sensorimotor system, and more specifically S1, is indeed involved in motor learning by AO [7]. We therefore support the assumption that the positive effect of both MVF and VT on the untrained hand is based on visuomotor inputs and sensorimotor inputs received by AO. This is further supported by results indicating that regions of visual attention and the integration of visual and somatosensory information, such as the secondary visual cortex (V2) and the anterior intraparietal sulcus (aIPS), are active during both MVF and VT [22, 40, 48]. Observed more closely, the aIPS, as the human homologue of the AIP in nonhuman primates, is part of the AON and links V2 and the PMC during the visuomotor processing [18, 61–63]. The PMC is densely connected to the hand representation area of M1 [64] and thus crucial for the combination of both external sensory signals and learned motor behavior in order to interact with the hands in the peripersonal space [65, 66]. Consequently, the involvement of the PMC during AO results in a cortical formation of new motor memory traces in M1 [29–31] that is consistent with studies on AO which demonstrated the vital importance of the AON in observational learning of new guitar chords [24, 25] and motor skills after stroke [26]. Taken together, the *first* and *second* experiments provide evidence that the increased motor outcome of the untrained hand is a result of the received visuomotor and sensorimotor inputs by AO, which are mediated via both a mirror and action-related video clips.

**Figure 5:**
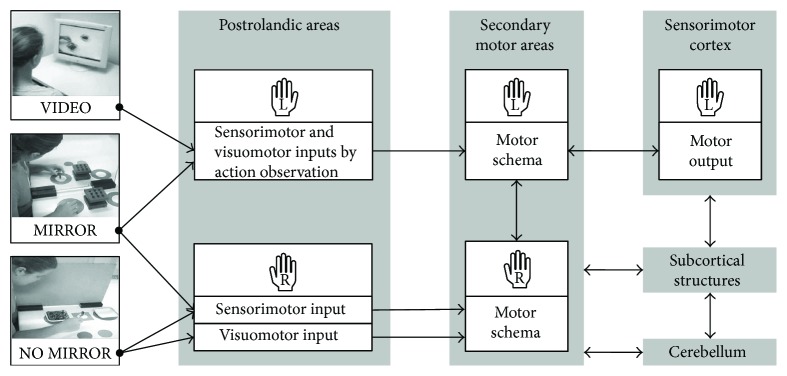
Proposed conceptual framework for acquired motor competence of the untrained (left) hand as a result of different network inputs.

Considering the effectiveness of MVF (MIRROR) and VT (VIDEO) with respect to the untrained hand, there is an obvious difference in favor of MVF (see Figure 4). Hence, visuomotor and sensorimotor inputs by AO alone are not a sufficient explanation for the positive effect during training with MVF. Indeed, performance improvements of the untrained hand after unilateral skill training without mirror feedback (NO MIRROR) are due to the intermanual transfer, which is accompanied by changes in interhemispheric interactions between left and right motor cortices via the corpus callosum [65, 67, 68]. A complete section of the corpus callosum stops or greatly decelerates the intermanual transfer [69]. However, studies using MVF found that more interregional than interhemispheric interactions between primary motor cortices are crucial for the performance improvements [37, 38]. These results were confirmed by a case study that reported MVF-induced performance improvements in patients despite a callosal section [42]. Moreover, excitability in motor-related areas contralateral to the untrained hand is facilitated by training with MVF [41, 70, 71]. It is likely that during MVF training, received inputs via AO are combined with parallel-mediated sensorimotor inputs by the actual execution with the training hand (see Figure 5) that is different to VT where only information is received via the observed video clips. Therefore, concerning the effectiveness of MVF, we suggest that MVF provides a more holistic training due to the combination of parallel received inputs via the AON and sensorimotor inputs by the actual execution.

### 4.2. The Possibility of an Increased Training Effectiveness by Combining Mirror Visual Feedback and Video Therapy

In our *third* experiment, we combined VT and MVF (VIDEO + MIRROR) to further boost the effectiveness of MVF by AO. To our knowledge, there is no study that has already attended this issue. We found that effectiveness of VT in addition to MVF is not substantially greater than VT in addition to training without mirror feedback (VIDEO + NO MIRROR) nor MVF alone (MIRROR) (see Figures 3 and 4). This result clearly supports the idea that both VT and MVF rest upon the AO concept mediating information about an action via the same pathways of the AON. Therefore, we argue that there is a striking resemblance between received video-based and mirror-based inputs (see Figure 5). This resemblance of received information is reflected by the similar effectiveness of VT in combination with MVF in contrast to MVF alone (see Figure 4). However, the controlling condition of the *third* experiment, which was a combination of VT and immediate execution without mirror feedback (VIDEO + NO MIRROR), showed a greater effectiveness in comparison to the sole training without mirror feedback (NO MIRROR) and a comparable effectiveness to MVF alone (see Figure 4). It is likely that the received inputs during the observation of action-related video clips are complemented by sensorimotor experience during the immediate execution with the training hand. Therefore, the motor schema is fed by visuomotor and sensorimotor inputs of the untrained hand during VT and sensorimotor inputs during the immediate action execution with the training hand (see Figure 5). These inputs are also processed during MVF. Thus, we suggest that motor competence of the untrained hand can be acquired both by parallel as well as directly consecutive visuomotor and sensorimotor inputs. However, combined methods in the third experiment, although their trainings sessions lasted twice as long as training sessions in all previous experiments, did not substantially increase effectiveness in comparison to MVF. Thus, the combination of MVF and VT turns out to be less effective looking from a clinical perspective.

To sum up the present study, we offer a conceptual framework (see Figure 5) that highlights our suggestions regarding acquired motor competence of the untrained hand as a result of different network inputs. Video clips that show a task-specific motor performance with the target effector (VIDEO) convey visuomotor and sensorimotor information to the observer. This information is processed in postrolandic areas, which in turn trigger secondary motor areas to build up a motor schema consistent with the observed action of the target effector. By training with action-related video clips, the schema is refined before it is retrieved by the sensorimotor cortex during actual execution with the target effector. Subcortical structures such as the basal ganglia and the cerebellum have a determining influence on these processes [65]. During the execution in front of a mirror (MIRROR), visuomotor and sensorimotor inputs are mediated by mere observation of one's own action via a mirror. Due to the optical illusion during observation, the AON is fed by information that is consistent with the execution of the resting target effector. At the same time, sensorimotor inputs are conveyed via a further network as a consequence of the actual execution with the performing nontarget effector. As a result, a more holistic schema than by mere observational training (VIDEO) is built up, which in turn is reflected by a greater motor performance of the untrained hand after training with MVF. Consequently, the combination of action-related video clips and training in front of a mirror (VIDEO + MIRROR) has no additional effect on the motor outcome of the untrained hand since the same information is processed twice via the pathways of the AON. Therefore, an expanded training program including similar inputs is not effective means to introduce a new training stimulus over the course of four days. In contrast, during training without mirror feedback (NO MIRROR), a motor schema is built up by sensorimotor and visuomotor inputs of the nontarget effector. Both sensorimotor and visuomotor inputs contain information that is inconsistent with the execution of the resting target effector. Thus, the training effect for the untrained hand is smaller than by training with MVF. It is more probable that the acquired motor competence of the untrained hand is mediated via the intermanual transfer [65, 67, 68]. However, by combination of observational training with action-related video clips and training without mirror feedback (VIDEO + NO MIRROR), corresponding visuomotor and sensorimotor inputs of the target effector are processed additionally by AO. Therefore, by this combination, a comparable training effect to MVF is attainable, although the training lasts twice as long.

## 5. Limitations and Clinical Implications

One limitation of this study is that it demonstrates results from healthy volunteers. Considering this, clinical implications addressing patients that suffer from motor deficits can only be drawn with reservation. Furthermore, the motor outcome of the untrained hand was only measured by means of a behavioral parameter. We did not present any neuronal correlates that could possibly elucidate the causal background of the training-induced increase of motor performance on the untrained hand. Any functional mechanisms, that is, underlying networks, and training-induced changes or modulations could only be presumed and argued with reference to comparable studies. We did not conduct a kinematic examination of muscle activity or trajectory. Therefore, implications regarding the quality of the observed performance increase are not thoroughly conclusive. However, we mainly focused on the practicability of the deployed methods.

Considering that our results from the *third* experiment provide a clear indication that the combination of VT and MVF under the defined conditions is not beneficial, we conclude that the application is not suitable in a clinical context. Future studies therefore should focus on a training that provides a further input to the motor control network besides AO. Thus, an additional increase in the effectiveness of the motor outcome could be achieved.

## 6. Conclusion

The results at hand demonstrate that AO induces a boosting effect on the motor outcome of the untrained hand during training with both MVF and VT. We therefore support that in both approaches, the pathways of the AON are employed to process visuomotor and sensorimotor inputs. Concerning MVF, our results suggest that additional sensorimotor inputs are processed in parallel by means of action execution with the nontarget effector. This is shown by the overall greater effectiveness of MVF training compared to VT alone. Our results indicate that the boosting effect by AO alone is limited. The doubling of AO during the training sessions by means of action-related video clips in combination with MVF did not substantially increase effectiveness of the training in comparison to MVF. It is more probable that the additional sensorimotor inputs of the nontarget effector are necessary to introduce a more effective training stimulus. In this context, our results also indicate that motor competence of the target effector benefits from additional AO when VT is combined with physical training of the nontarget effector in close succession. Therefore, from a methodological point of view, mere AO training is probably the method of choice to build up a precise schema of a target action within the initial stage of motor learning. On the contrary, MVF is the more effective approach to increase the motor performance of the untrained hand in the further process of motor learning because of the combination of AO and physically received sensorimotor inputs.

## Abbreviations

AIP: Anterior intraparietal area
aIPS: Anterior intraparietal sulcus
AO: Action observation
AON: Action observation network
fMRI: Functional magnetic resonance imaging
IPL: Inferior parietal lobule
IFG: Inferior frontal gyrus
M1: Primary motor cortex
MNS: Mirror neuron system
MVF: Mirror visual feedback
PMC: Premotor cortex
S1: Primary somatosensory cortex
S2: Secondary somatosensory cortex
STS: Superior temporal sulcus
V2: Secondary visual cortex
VT: Video therapy.
